# The integrated stress response in cancer: mechanisms of tumor adaptation and therapeutic targeting

**DOI:** 10.1042/BST20250133

**Published:** 2026-04-27

**Authors:** Elias Maldonado, Emily McIsaac, Josie Ursini-Siegel

**Affiliations:** 1 Division of Clinical and Translational Research, McGill University, Montreal, Canada; 2 Lady Davis Institute for Medical Research, McGill University, Montreal, Canada; 3 Gerald Bronfman Department of Oncology, McGill University, Montreal, Canada; 4 Department of Biochemistry, McGill University, Montreal, Canada

**Keywords:** ATF4, cancer, eIF2alpha, Integrated Stress Response

## Abstract

Cancer cells face continual stressors, which they must overcome to proliferate and survive in the body. Under these conditions, essential biochemical pathways are disrupted, contributing to various stress responses that either promote adaptation and survival or eventual cell death. The evolutionarily conserved integrated stress response (ISR) is a key adaptive mechanism that transiently rewires the transcriptome and translatome in response to various stressors. While the ISR is activated in healthy cells under moderate stress, cancers especially rely on this pathway to overcome harsh conditions experienced during tumor growth and metastasis. We explore the pro-tumorigenic role of the ISR, along with the upstream stress-sensing kinases that activate it. These include protein kinase R-like endoplasmic reticulum kinase, general control non-derepressible 2, double-stranded RNA-dependent protein kinase, and heme-regulated eukaryotic translation initiation factor 2α kinase (HRI), which initiate an ISR in response to diverse stressors by phosphorylating their shared substrate, eukaryotic initiation factor-2α. An in-depth understanding of the pro-survival functions of the ISR and the contexts in which it is pro-tumorigenic is necessary to leverage the ISR as a therapeutic strategy.

## Introduction

Aggressive cancers face multiple sources of cellular stress that must be overcome to establish and maintain malignancy. As tumors grow, their expanding mass often outgrows their blood supply, leading to oxygen deprivation, nutrient limitation, and difficulty meeting the energetic demands of rapidly proliferating cancer cells [1]. Cancer cells experience endoplasmic reticulum (ER) stress if they cannot maintain the energetic requirements that support increased rates of protein synthesis and folding [2,3]. The increased metabolic rate of cancer cells also increases production of reactive oxygen species (ROS), leading to oxidative stress if their antioxidant systems cannot buffer these ROS molecules [2,4]. These, among other selective pressures faced by cancer cells, activate a stress adaptation pathway called the integrated stress response (ISR).

**Figure 1:**
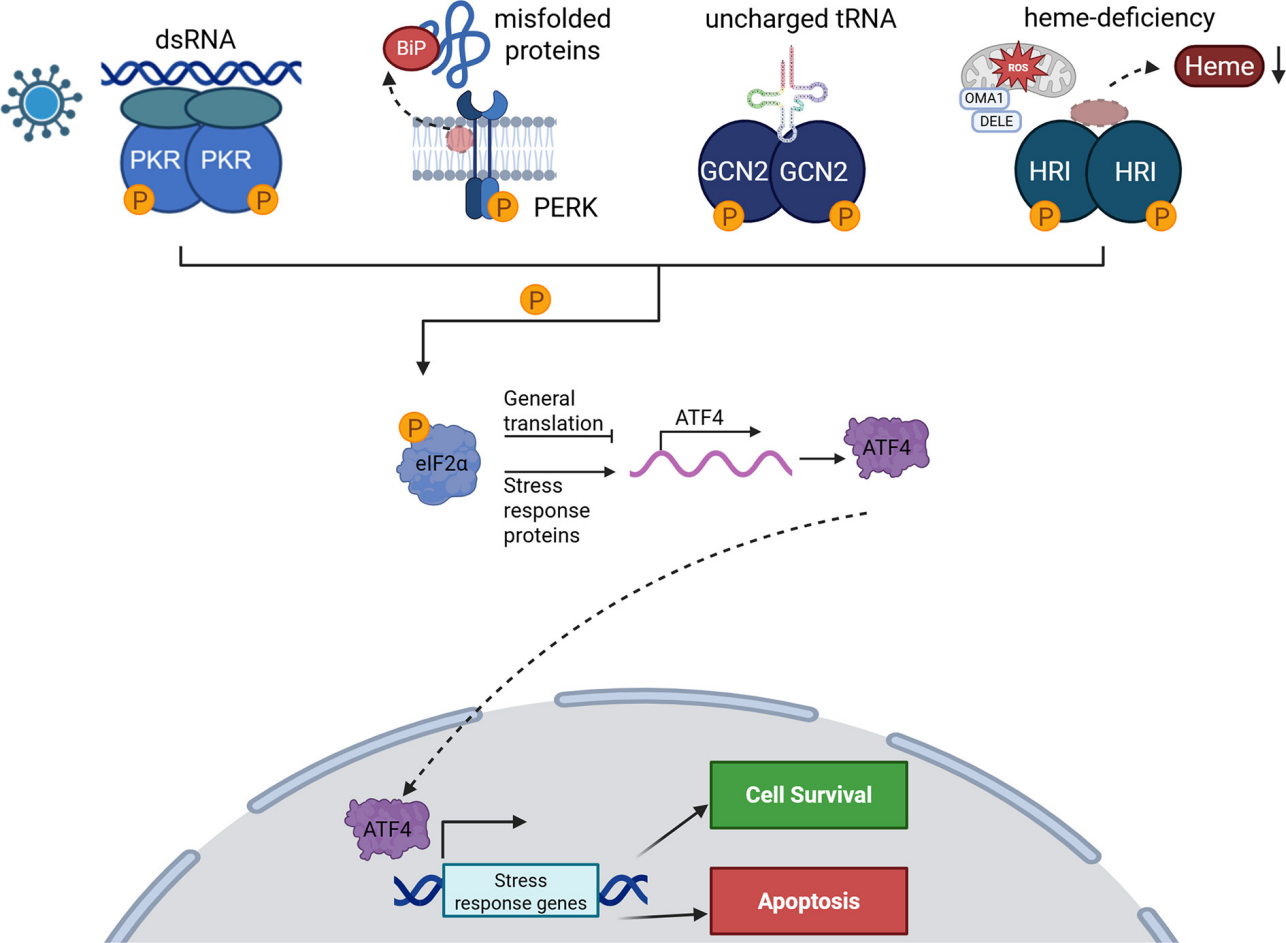
The integrated stress response The integrated stress response (ISR) is activated by one of four stress kinases, PERK, GCN2, PKR, and HRI, which phosphorylate Ser51 of eIF2α to suppress global protein synthesis while promoting selective cap-independent translation of stress-responsive transcripts, such as ATF4. ATF4 drives transcriptional programs that either promote survival and restore cellular homeostasis or trigger cell death, depending on the severity of the stress. Created in BioRender. McIsaac, E. (2026) https://BioRender.com/0s61xmt.

**Figure 2:**
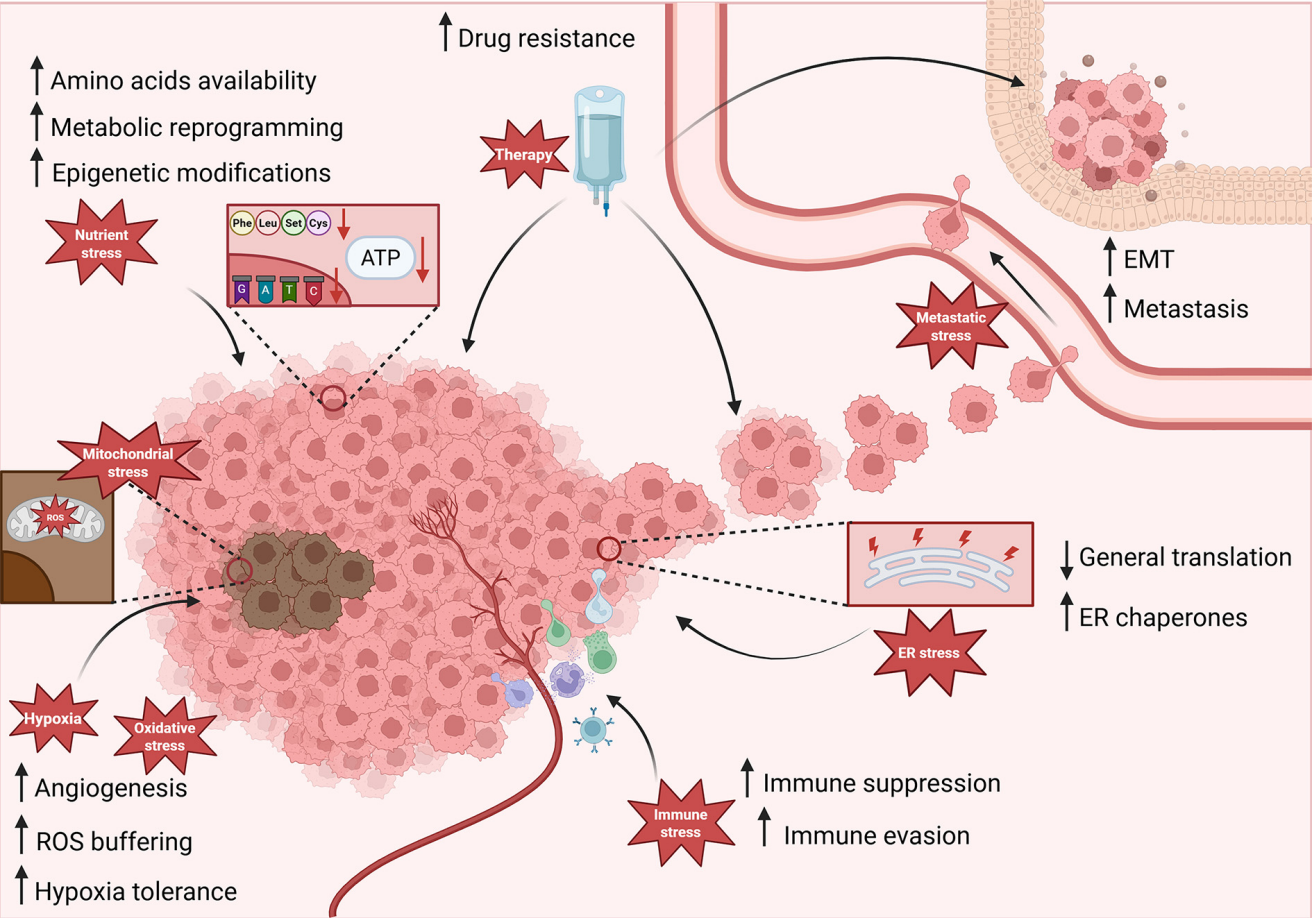
The modulation of stress responses by the ISR in cancer Cancers experience many kinds of stress, such as hypoxia, oxidative stress, mitochondrial stress, nutrient deprivation, therapeutic stress, metastatic stress, ER stress, and immunological stress. The ISR is engaged downstream of multiple stressors in cancer cells to engage distinct adaptive responses that support their survival and eventual progression to malignancy or therapy resistance. Created in BioRender. McIsaac, E. (2026) https://BioRender.com/0s61xmt.

Present in all eukaryotes, the ISR allows cells to respond to diverse stressors through the action of four stress-sensing kinases that converge upon the eukaryotic initiation factor-2α (eIF2α) as a common substrate. These include: (1) protein kinase R-like endoplasmic reticulum kinase (PERK), (2) general control non-derepressible 2 (GCN2), (3) double-stranded RNA-dependent protein kinase (PKR), and (4) heme-regulated eukaryotic translation initiation factor 2α kinase (HRI) [5–7]. eIF2α kinases are specialized to respond to a unique stress; PERK is activated during ER stress, GCN2 is activated by amino acid deficiency, PKR is activated by expression of intracellular dsRNAs or viral infection, and HRI is activated by heme depletion and mitochondrial dysfunction **(**Figure 1) [5–8]. These kinases phosphorylate Ser51 of eIF2α, a core component of the eIF2–GTP–Methionyl-tRNA ternary complex, which initiates cap-dependent mRNA translation [9]. In its unphosphorylated state, eIF2α enhances global protein synthesis by enabling the formation of the ternary complex to deliver the initiator Met-RNA to the 40S ribosome in a GTP-dependent manner [9]. Subsequently, eIF2B, the guanine exchange factor of eIF2, catalyzes GDP–GTP exchange to support subsequent rounds of initiation [9]. In contrast, phosphorylation of eIF2α at Ser51 converts eIF2 into a competitive inhibitor of eIF2B, greatly reducing GDP–GTP exchange, which depletes eIF2–GTP levels and blocks global mRNA translation initiation [5–7,9]. When global protein synthesis is halted, cells divert their energy from protein synthesis to stress adaptation mechanisms. This adaptive response is mediated, in part, by activating transcription factor 4 (ATF4), whose mRNA is translated specifically following eIF2α phosphorylation [7]. mRNA translation of ATF4 is tightly controlled by multiple upstream open reading frames (uORF). Under basal conditions, ribosomes translate uORF1 and rapidly re-acquire the ternary complex, enabling efficient re-initiation of translation at the downstream inhibitory uORF2. Since uORF2 overlaps out of frame with the ATF4 coding sequence and extends beyond the ATF4 start codon, its translation leads to ribosome termination and dissociation, preventing initiation at the ATF4 open reading frame [7]. In contrast, eIF2α phosphorylation reduces ternary complex availability, delaying re-initiation after uORF1. This delay allows scanning ribosomes to bypass the uORF2 start codon and instead initiate translation at the ATF4 coding region, promoting ATF4 protein synthesis in response to stress [7]. When translated, ATF4 induces expression of a plethora of stress response genes, allowing cells to alter their transcriptome to either adapt to the stress or be eliminated by apoptosis (Figure 1) [6,7]. This review will explore how cancers manipulate the ISR, both through ATF4-dependent and ATF4-independent mechanisms, to promote sustained growth and survival in harsh conditions. It will also address the potential to target the ISR therapeutically, shifting ISR signaling toward apoptosis in death-resistant cancer cells (Figure 2).

## Pro-tumorigenic roles of the ISR

### Endoplasmic reticulum stress

Protein synthesis and folding are tightly regulated processes to maintain ER homeostasis. Proteostasis requires adequate ATP and ER luminal calcium levels to support protein synthesis, chaperone function, and maintenance of an oxidizing ER environment for disulfide bond formation [10,11]. The rapid proliferation rate of cancer cells, combined with limited nutrient and oxygen availability in solid tumors, disrupts ER homeostasis and leads to an accumulation of misfolded proteins, which triggers the unfolded protein response (UPR) [10]. The UPR is controlled by three ER transmembrane proteins, PERK, inositol requiring enzyme 1 (IRE1), and activating transcription factor 6 (ATF6) [10]. While each induces transcriptional programs that increase ER chaperone activity, PERK also phosphorylates eIF2α, leading to a global inhibition of protein synthesis, preventing further accumulation of misfolded proteins [10,12]. In parallel, PERK-mediated activation of ATF4 induces adaptive stress response genes to restore proteostasis in cooperation with IRE1 and ATF6. If ER stress is sustained and severe, ATF4 signaling can shift the ISR from a pro-survival to a pro-apoptotic program [10].

PERK is beneficial for the survival of many tumors [13–20]. Oncogenic signaling increases the rates of protein synthesis and proliferation, making the UPR an essential adaptation to sustain tumor growth [21]. PERK-ATF4 signaling is increased across multiple cancers, with genetic or pharmacological PERK inhibition exacerbating ER stress, reducing tumor growth and promoting apoptosis [13,15–17,19]. Notably, PERK inhibition preferentially limits tumor growth *in vivo* while having minimal effects on cancer cells cultured under low-stress conditions, suggesting that stressors associated with tumor growth create a dependence on PERK for stress adaptation [13]. Numerous studies have demonstrated the ability of PERK to support cancer cell metastasis by inducing a transcriptional program that induces angiogenesis and an epithelial-to-mesenchymal transition (EMT) [22,23]. PERK signaling also contributes to an immunosuppressive microenvironment in response to hypoxia through pleiotropic mechanisms, including decreased MHC class I expression and antigen presentation by tumor cells [3,24]. Indeed, PERK loss in cancer cells restores type I IFN responses to promote anti-tumor T cell immunity [20]. PERK signaling has also been implicated in the survival of cancer cells following chemotherapy, promoting the emergence of dormant or residual-disease cancer cells and eventual tumor recurrence [18,25,26]. Although PERK-UPR signaling is often co-opted to support tumor cell survival, cancers must tightly regulate this pathway to achieve an optimal ISR output, as excessive PERK activity can promote ATF4-dependent induction of the pro-apoptotic transcription factor C/EBP homologous protein (CHOP) when the stress is prolonged and severe [10,13]. For example, activation of the PERK/ATF4/CHOP pathway promotes cancer cell death and limits tumor growth in response to several therapies, suggesting that therapeutic hyperactivation of PERK signaling or inhibition of ER stress resolution may shift tumors from an adaptive to a pro-apoptotic ISR [27–29]. However, chronic and mild ER stress permits CHOP to act as a stress sensor that promotes cellular adaptation and restoration of protein synthesis [30]. These studies underscore the context-dependent and dynamic nature of ISR signaling in tumorigenesis.

### Nutrient stress

Nutrient availability is tightly regulated by many convergent pathways to protect cellular fitness by ensuring adequate amino acid bioavailability [31]. The main sources of amino acids are via *de novo* synthesis pathways or through the intake of dietary amino acids via transporters [31]. Beyond their role in protein synthesis, amino acids aid in crucial pathways including nucleic acid synthesis, epigenetic modification, energy production, oxidative stress management, and regulating cell death [32]. Therefore, perturbation of amino acid availability simultaneously impacts several homeostatic pathways. Under anabolic conditions, cancer cells up-regulate amino acid uptake and *de novo* synthesis to promote sustained proliferation and survival [32]. However, under catabolic conditions, including decreased nutrient availability, hypoxia, or other conditions that increase energetic demand (i.e., survival in circulation), cancer cells must rapidly transition to utilizing nutrients, including amino acids, to support their survival. When external sources of amino acids are limiting or are rapidly consumed, the accumulation of uncharged tRNAs triggers the activation of GCN2, which induces an ISR in cancer cells. Under these conditions, the ISR aids in tumorigenesis by regulating nutrient availability. For example, a GCN2-driven ISR is activated during myeloma progression and contributes to malignant cancer cell survival [33].

In Myc-driven cancers, elevated rates of protein synthesis increase their metabolic demands. When they are unmet, high Myc levels activate both GCN2 and PKR to induce an ISR, establishing a negative feedback loop that limits Myc translation [34]. This restrains Myc-induced proteotoxic stress by simultaneously regulating amino acid metabolism and translational control, suggesting an ATF4-dependency that renders Myc-driven tumors susceptible to inhibition of the ISR [35,36]. Similarly, the oncogene AMMDC, which is transcriptionally induced by ATF4 and Myc, promotes metabolic reprogramming by inducing one-carbon and lipid metabolism to promote cancer cell survival [37]. Other proteins, such as the AAA+ ATPase VCP/p97, play a significant role in coordinating protein quality control pathways. Indeed, VCP/p97 acts in a coordinated manner with GCN2 to preserve proteostasis and metabolic stability when nutrients are scarce, highlighting a novel stress adaptation module for tumor cells [38]. Thus, oncogenes can alter tumor metabolism through the ISR to support cell survival.

In response to nutrient deprivation, cancer cells also up-regulate amino acid transporters through the ISR. Depletion of GCN2 and ATF4 in prostate cancer cells reduces expression of amino acid transporters and cellular amino acid levels, inducing cell death [39]. Other amino acid transporters are also regulated by ATF4 and contribute to tumorigenesis, including SLC38A2 and LAT1, which are transporters for neutral amino acids (glutamine, alanine, and serine) and large neutral amino acids (leucine, valine, and phenylalanine), respectively [40,41]. Together, these studies highlight the importance of an ISR in supporting cancer progression by increasing expression of amino acid transporters to limit proteotoxic stress.

An ISR is also central to the ability of cancer cells to restore amino acid levels through *de novo* synthesis. Activation of an ISR promotes *de novo* asparagine synthesis, an amino acid that plays many roles in the cell beyond protein synthesis, as it can feed into the citric acid cycle or be used to generate nucleotides [42]. Cancer cells with increased ISR activity up-regulate asparagine synthetase, the rate-limiting enzyme in asparagine synthesis, promoting cell growth and survival [43–46]. The ISR also impacts serine biosynthesis by increasing the expression of PHGDH (phosphoglycerate dehydrogenase), which promotes one-carbon metabolism and epigenetic reprogramming [47–49]. Additionally, long non-coding RNAs regulated by ATF4 alter serine biosynthesis to promote tumor cell survival [50]. Together, these studies highlight the many mechanisms by which the ISR can aid cancer cells to overcome nutrient stress and survive harsh metabolic conditions.

### Oxidative stress

Oxidative stress is defined as an imbalance between ROS and antioxidant defenses in favor of ROS, leading to damage to DNA, lipids, or proteins [4]. Tumor cells experience chronically and moderately elevated, but sub-lethal, ROS levels due to their high metabolic rates through increased oxidative phosphorylation and nonmitochondrial ROS sources. Cancer cells respond by up-regulating their antioxidant machinery to adapt to and exploit chronically elevated ROS signaling [4]. The transcription factor NRF2, a master regulator of the antioxidant response, is classically activated by oxidative stress but is also transcriptionally induced downstream of the ISR via ATF4 [51]. NRF2 increases glutathione (GSH) levels by inducing expression of the glutathione synthesis machinery [51]. The ability of cancer cells to maintain their GSH pools relies on increasing uptake of extracellular cystine, the oxidized dimer of cysteine and rate-limiting amino acid for GSH synthesis, through the xCT cystine/glutamate antiporter (SLC7A11) [52]. In this regard, ATF4 not only up-regulates SLC7A11 expression to support GSH synthesis but also expands the ROS scavenging capacity by inducing expression of additional antioxidants, including components of the thioredoxin pathway [51–53]. By increasing intracellular cystine, the xCT cystine transporter ensures tumor cell survival under conditions that induce oxidative stress [54–56]. In triple negative breast cancer (TNBC) models, ATF4 drives expression of multiple GSH biosynthetic genes to promote radioresistance [57]. In colorectal cancers, PERK signaling induces cytoprotective antioxidant responses through ATF4, and PERK inhibition sensitizes them to ferroptotic cell death [14,16]. In addition to increasing ROS buffering, ATF4 also up-regulates the ER chaperone HSPA5, which binds and stabilizes glutathione peroxidase 4 (GPX4), a key enzyme that protects cells from ferroptosis by detoxifying harmful lipid peroxides [58]. Collectively, these studies illustrate how ISR signaling restores redox balance in cancer cells experiencing increased oxidative stress to support their growth and survival.

## Oncogenic stress

Most cancers are driven by oncogenes that bypass regulatory checkpoints, leading to uncontrolled proliferation and oncogene-induced stress, such as DNA damage and limited nutrient availability [59]. Interestingly, oncogenic signaling frequently engages the ISR to buffer oncogene-induced stress, with reciprocal regulation observed between ISR activity and oncogene expression. For example, Myc expression is influenced by ISR signaling, while MET translation is itself enhanced following eIF2α phosphorylation, promoting invasive growth under various stresses [36,42,60,61]. Conversely, oncogenic KRAS activates the ISR during nutrient stress by inducing ATF4 and its transcriptional program through coordinated regulation by the AKT and NRF2 pathways [62]. In KRAS mutant tumors, KRAS rewires the cellular response to nutrient stress by engaging an ATF4-dependent transcriptional program to support amino acid homeostasis [62]. This requires AKT signaling to sustain NRF2 activity, which tunes the magnitude of ATF4 target gene expression, enabling an adaptive program that increases amino acid uptake and *de novo* synthesis to support protein synthesis and survival during nutrient stress [62]. However, excessive NRF2 activation, such as in the context of KEAP1 loss, shifts the ATF4-dependent response toward apoptosis, highlighting the context-dependent nature of oncogenic pathways and ISR signaling in cancer [62]. This context-dependent role of the ISR extends to the ISR kinases themselves, as PERK can behave as a tumor suppressor or tumor promoter in BRAF-driven melanomas in a dose-dependent manner [63]. Thus, while oncogene-induced cellular stress commonly engages the ISR, its effects on cell fate are highly context-dependent.

## ATF4-independent mechanisms of action

While eIF2α kinases are best known for phosphorylating eIF2α to increase ATF4 function, they also exert equally important ATF4-independent effects on stress adaptation. Like ATF4, other mRNAs are regulated by ribosomal bypass of upstream inhibitory ORFs, such as the oncogene MET [7,61]. Other examples include two immune checkpoint genes, PD-L1 and CD155, whose rates of mRNA translation are increased during an ISR by inhibitory uORF skipping, promoting tumor immune evasion [64,65]. Similarly, stemness inducers, including NANOG, SNAIL, and NODAL, show increased ISR-dependent mRNA translation of alternative 5′ UTRs in response to hypoxia, which promotes breast cancer plasticity [66].

In specific contexts, global inhibition of protein synthesis alone supports tumorigenesis. As mentioned previously, Myc-driven tumors must engage adaptive mechanisms that reduce their high rates of global protein synthesis to prevent proteotoxic and metabolic stress. In this regard, Myc and ATF4 increase transcription of 4E-BP1, a negative regulator that prevents eIF4F assembly and initiation of cap-dependent mRNA translation. In these tumors, 4E-BP1 acts as a rheostat to maintain optimal rates of protein synthesis [35]. Similarly, APC-deficient colorectal tumors rely on an ISR to restrain Myc levels. Disrupting the ISR unleashes uncontrolled levels of Myc translation and paradoxically suppresses tumor growth by depleting amino acid and nucleotide pools [34]. Thus, eIF2α kinases and the ISR can also support tumor survival through ATF4-independent mechanisms.

Finally, individual eIF2α kinases have unique substrates beyond eIF2α. These non-ISR-related functions are best studied for PKR, which phosphorylates several components of the NF-κB (nuclear factor kappa-light-chain-enhancer of activated B cells) pathway to induce NF-κB signaling and promote inflammatory responses in response to viral infection and/or activation by endogenous dsRNAs [67]. Moreover, PKR can promote immune evasion by increasing PD-L1 expression through the PKR/STAT1/IRF1 pathway and by inducing immunosuppressive TGF-β signals [68–71]. Paradoxically, PKR can also sensitize tumors to immunotherapies by targeting its interactors, ADAR1 and PACT, leading to detrimental amounts of PKR activation and anti-tumorigenic inflammation [72–74]. In addition, NRF2 is also a direct substrate for PERK, whereby NRF2 phosphorylation increases its stability to ensure a robust cytoprotective response to oxidative insults [21]. Though other non-ISR pathways likely exist downstream of eIF2α kinases, many substrates have yet to be identified, and further study of these functions in the context of cancer is necessary.

## Non-canonical ISR states

Like ATF4-independent ISR functions, the ISR can also adopt non-canonical states. Under acute stress, ISR activation via eIF2α phosphorylation suppresses global translation while selectively promoting translation of stress response genes that remodel the transcriptome [5–7,9]. Following stress resolution, ATF4 and other ISR effectors restore translation through induction of genes such as GADD34, which dephosphorylates eIF2α via PP1, whereas unresolved stress triggers transcription of pro-apoptotic genes [75]. However, multiple studies have described non-canonical ISR states in which phosphorylated eIF2α is uncoupled from global translational repression and ATF4 translation [75–77]. For example, under chronic ER stress, mouse embryonic fibroblasts sustain ISR signaling while partially restoring translation through eIF4-independent but eIF3-dependent translation initiation, enabling survival [75]. In addition, senescent cells suppress the ISR by failing to translate ATF4 despite elevated basal levels of eIF2α phosphorylation, reflecting a fundamentally reduced translational capacity rather than specific ISR engagement [76]. The reduced ribosome abundance and low translational demands of senescent cells limit ternary complex utilization and impair bypass of the inhibitory uORF2 to limit ATF4 translation even under conditions that would normally activate an ISR [76]. The consequence of this maladaptive ISR in senescent cells instead favors remodeling of the senescence-associated secretory phenotype, amplifying sustained inflammatory signals [76]. ATF4 transcription can also be repressed following UV radiation despite eIF2α phosphorylation [77]. Similarly, some dormant cancer cells can specifically degrade ATF4 during ISR activation, since ATF4 induction during ER stress accelerates recovery of protein synthesis, which increases their sensitivity to chemotherapy-induced cell death [78]. Thus, dormant cancer cells that suppress this ATF4-driven recovery maintain low rates of mRNA translation and are better able to evade chemotherapy. Although these non-canonical ISR states have largely been described in non-transformed cells, they highlight contexts that may exist within cancer where ISR markers and outputs are uncoupled, underscoring the need to better define ISR context specificity in cancer and its therapeutic implications.

## Therapeutic strategies

### Inhibiting the ISR

There is robust evidence that the ISR is activated by therapies, granting cancer cells stress adaptation mechanisms that allow resistance to chemotherapies, radiotherapy, and targeted therapies [45,47,57,66,79–83]. Since the ISR is crucial for the survival of chronically stressed cancer cells, inhibiting the ISR can impair tumor growth in preclinical models of cancer. ISRIB is a prominent small molecule inhibitor of the ISR, as it maintains the activity of the eIF2 complex despite the phosphorylation of eIF2α, allowing mRNA translation to proceed [84,85]. ISRIB prevents adaptive responses in cancer cells, limiting their ability to maintain mitogenic signaling, buffer ROS, maintain stemness/plasticity, survive chemotherapy, and evade the immune system, all leading to reduced tumor growth and increased cytotoxicity in preclinical models [15,64–66,80]. Inhibiting eIF2α kinases also limits tumor growth in several studies. Selective PERK inhibitors (HC-5404, GSK2606414, GSK2656157, and AMGEN44) limit tumor growth by sensitizing tumors to ferroptosis, inducing cancer-specific cellular senescence, killing dormant metastases, and synergizing with targeted therapies to prevent PERK-driven resistance mechanisms [15–18,26]. Of these, only HC-5404 has been evaluated in a phase I clinical trial (NCT04834778), with encouraging preliminary efficacy in patients with advanced solid tumors. One GCN2 inhibitor (APL-4098) is currently recruiting for a phase I clinical trial in acute myeloid leukemia patients (NCT06372717), while other GCN2 inhibitors (A-92 and GCN2ib) have shown potential preclinically in Myc-driven cancers [33,34,39]. Although PKR inhibitors exist (C16 and 2-AP), they lack sufficient specificity and have rarely been studied in preclinical cancer models [86,87]. Interestingly, PKR activity in tumors limits the efficacy of oncolytic viruses [88]; however, engineering them to impair PKR expression and/or activity greatly improves the anti-tumor immune responses initiated by oncolytic viruses [69–71].

However, many eIF2α and tyrosine kinase inhibitors paradoxically activate other eIF2α kinases, leading to sustained ISR, which complicates potential therapeutic strategies [89,90]. Compensation between the eIF2α kinases to maintain eIF2α phosphorylation in response to genetic silencing or pharmacological inhibition of individual eIF2α kinases has also been observed, further complicating therapeutic strategies to inhibit the ISR [34,88,91–93]. Thus, more advancements are necessary to identify efficient compounds and drug combinations that can inhibit the ISR without these undesirable secondary effects that limit their efficacy.

### Hyperactivation of the ISR and targeting ISR-driven vulnerabilities

While chronic and moderate elevation of the ISR supports tumorigenesis, identifying therapies that hyperactivate a prolonged ISR to induce its apoptotic arm may also be a promising strategy, particularly in combination with standard therapies. Salubrinal, an inhibitor of eIF2α phosphatases, sensitizes HER2+ breast and gastric tumors to trastuzumab treatment due to ATF4-dependent expression of CDK inhibitors [94]. Additionally, several compounds were identified to induce mitochondrial dysfunction through activation of the OMA1/DELE1/HRI pathway, leading to downstream pro-apoptotic signaling through ATF4/CHOP, sensitizing cancers to cisplatin treatment [8,95,96]. While ISR agonists show some success at promoting ISR-induced apoptosis, many studies find that polarization of the ISR pathway toward apoptosis is possible by inducing extreme stress via other targeted therapies. For example, chemotherapies and several targeted therapies that cause severe oxidative or ER stress induce apoptosis through an ISR [27,29,54,97–99]. While the ISR supports tumor growth and therapy resistance [45,47,79,81,82,100], some cancer cells may become reliant on the ISR, exposing a metabolic vulnerability to efficiently kill the cancer cells while sparing normal cells [19,44,45,47,79,81,82,100,101]. Therefore, while the ISR provides cancer cells with stress adaptation mechanisms, it also renders them vulnerable to other stressors and to hyperactivation of the ISR.

## Conclusion and future directions

In this review, we highlighted emerging insights into the tumor-supporting functions of the integrated stress response (ISR) across diverse cancer contexts. While the roles of GCN2 and PERK in controlling tumor growth or therapeutic response are better characterized, studies examining the impact of PKR or HRI in the oncology setting are limiting. Regardless, a growing body of literature demonstrates that ISR activation enables tumor cells to adapt to the energetic, proteotoxic, nutrient, and oxidative stresses inherent to tumorigenesis, thereby enhancing cellular fitness and contributing to growth, survival, and metastatic fitness. These adaptive outcomes often depend on maintaining a ‘Goldilocks’ level of ISR activity—one that is sufficiently robust to restore homeostasis through ATF4-dependent transcriptional programs yet not so severe as to trigger cell death. This delicate balance has positioned the ISR as a potential therapeutic target: attenuating ISR activity can blunt pro-survival stress adaptations, whereas hyperactivating the pathway can overwhelm its buffering capacity and drive apoptosis. Although multiple promising preclinical compounds illustrate the translational potential of ISR-targeted therapies, further work is needed to define context-dependent ISR states and combat compensatory eIF2α kinase activation.

## Perspectives

- Cancers rely on pro-tumorigenic ISR signaling, engaging both ATF4-dependent and ATF4-independent mechanisms to overcome diverse stressors, such as ER stress, nutrient stress, and oxidative stress, allowing them to survive and grow under harsh conditions and resist therapy.
- Pro-tumorigenic ISR signaling relies on halting global protein synthesis while achieving an optimal balance of selective expression of ATF4-dependent genes to promote stress adaptation and cell survival by re-establishing homeostasis of important biochemical pathways during stress.
- A deeper understanding of the context in which the ISR is pro- and anti-tumorigenic, along with further insight into the heterogeneous and non-canonical ISR states in cancer, is necessary before the ISR can be leveraged as a novel therapeutic strategy to counteract adaptive properties of aggressive cancers and push cancer cells toward apoptosis.

## Open Access

Open access for this article is provided via our Subscribe to Open programme, made possible through the support of our subscribing institutions. Learn more here: https://portlandpress.com/pages/open_access_options_and_prices#conditional.

## Abbreviation

4E-BP1: eukaryotic translation initiation factor 4E-binding protein 1
ATF4: activating transcription factor 4
ATF6: activating transcription factor 6
CDK: cyclin dependent kinase
CHOP: C/EBP homologous protein
eIF2: eukaryotic initiation factor 2
eIF2α: eukaryotic initiation factor 2 alpha
EMT: epithelial mesenchymal transition
ER: endoplasmic reticulum
GCN2: general control non-derepressible 2
GPX4: glutathione peroxidase 4
GSH: glutathione
HER2: human epidermal growth receptor 2
HRI: heme-regulated eukaryotic translation initiation factor 2α kinase
HSPA5: heat shock protein family A member 5
IRE1: inositol requiring enzyme 1
ISR: integrated stress response
ISRIB: integrated stress response inhibitor
NF-κβ: nuclear factor kappa-light-chain-enhancer of activated B cells
NRF2: nuclear factor erythroid 2-related factor 2
ORF: open reading frame
PD-L1: programmed death ligand 1
PERK: protein kinase R-like endoplasmic reticulum kinase
PHGDH: phosphoglycerate dehydrogenase
PKR: double-stranded RNA-dependent protein kinase
ROS: reactive oxygen species
TNBC: triple negative breast cancer
UPR: unfolded protein response
UTR: untranslated region
